# Asynchronous Remote Communication as a Tool for Care Management in Primary Care: A Rapid Review of the Literature

**DOI:** 10.5334/ijic.6489

**Published:** 2022-08-11

**Authors:** Aïna Fuster-Casanovas, Josep Vidal-Alaball

**Affiliations:** 1 Unitat de Suport a la Recerca de la Catalunya Central, Fundació Institut Universitari per a la Recerca a l’Atenció Primària de Salut Jordi Gol i Gurina, Sant Fruitós de Bages, Spain; 2 Health Promotion in Rural Areas Research Group, Gerència Territorial de la Catalunya Central, Institut Català de la Salut, Sant Fruitós de Bages, Spain; 3 Faculty of Medicine, University of Vic-Central University of Catalonia, Vic, Spain

**Keywords:** primary health care, remote consultation, patients, quality indicators, patient satisfaction, health systems plans

## Abstract

**Aim:**

To review the available evidence on asynchronous communication models between primary care professionals and patients in different countries around the world in order to analyse the added value that this model brings to patients and professionals.

**Design:**

A rapid literature review was conducted using the World Health Organisation guidance to include a variety of studies on the concept of asynchronous communications between primary care and patients in different countries.

**Data sources:**

The search for articles was carried out in PubMed and Google Academics and with the contribution of telemedicine experts from the Catalan Institute of Health.

**Selection of studies:**

The review included 271 articles. The inclusion criteria were: publications from 2010 onwards, in English, Spanish or Catalan, focused on asynchronous communications between primary care professionals and patients to improve patient management. After discarding duplicates and applying the exclusion criteria (255 articles), 16 were included for further review.

**Data extraction:**

The rapid literature review was conducted by an evaluator; detecting 5 main general themes: reduction of face-to-face visits, available services and most frequent uses, characteristics and perceptions of primary care professionals, characteristics and perceptions of users, and barriers and facilitators for the implementation of asynchronous teleconsultation.

**Results:**

A total of sixteen studies were included, including seven quantitative studies, seven qualitative studies and two mixed studies.

**Conclusions:**

The high degree of satisfaction of both users and professionals, the outbreak of COVID-19 and the effectiveness and efficiency of asynchronous remote communications are key factors for the implementation and improvement in the management of the different healthcare systems across the world.

## Introduction

Over the last decade, telemedicine services have been introduced in the health systems of different industrialised countries [1]. One example is the introduction of asynchronous remote communications between professionals and patients in primary care centres. In Catalonia, a region of Spain, this tool is called eConsulta, being a part of the public health IT system [2,3,4].

In this context, patient acceptance is a key indicator in the system to encourage the use of eHealth, as otherwise the services offered become redundant and expensive [5]. In order to establish telemedicine as a routine tool, acceptance by professionals is also necessary. For that, it is necessary to know what the main factors are that create opposition. Scott C. et al. identify these possible barriers: technical difficulties (11%), resistance to change (8%), co-payment (5%), patient age (5%) and patient education level (5%) [6].

With the expansion of the pandemic caused by the SARS-CoV-2 coronavirus, the development of information and communication technologies has been essential to support the provision of safer and more effective health care, reducing the risk of direct person-to-person exposure and improving the management of care flows in health systems [7,8,9]. Within this context, asynchronous teleconsultation has helped to maintain communication between health professionals and patients at a crucial time when it was necessary to avoid trips to primary care centres [10]. Solans O. et al. in Spain show how the volume of use of this digital solution during the period before and during COVID-19 tripled in the first 3 months of the pandemic. This tool, in a context of primary care overcrowding, has improved care management [11].

The aim of this article is to undertake a rapid review of the scientific literature to analyse the value of asynchronous telematic communications between primary care professionals and patients in health systems in different countries.

## Materials and methods

We carried out a rapid review of the scientific literature following the World Health Organisation’s (WHO) Guidelines for Rapid Reviews [12]. This methodology was developed to establish a transparent and scientific, detailed and reproducible method. Although systematic reviews are considered to be the best option for health decision management, rapid reviews emerge as a response to the long time and resources involved in clinical decision making and policy design. In this way, rapid reviews use a methodology similar to systematic review, but with variations in the development, allowing answers to be reached in a shorter time and with optimisation of resources. There is no consensus on which short cuts are taken in development, so rapid reviews are heterogeneous in respect of each other. In this context, the key principles of knowledge synthesis were respected including the objectives of the review, the prior definition of the selection criteria, the evaluation of the results and the presentation and synthesis of the results [12].

### Search strategy and data sources

A rapid review of the existing scientific literature was carried out in order to perform a qualitative analysis with deductive characteristics that encompassed the concept of asynchronous communications between primary care and patients. Studies from 2010 onwards were reviewed in the databases PubMed, Google Academics and the collaboration of telemedicine experts from the Catalan Institute of Health was requested. MeSH terms were used as search criteria adapted to each search engine, (e.g. *“primary care” AND “eConsult” OR “teleconsult” AND “patient” AND “quality” AND “satisfaction”*).

### Inclusion and exclusion criteria

The search identified those studies that, in the title, abstract or text, included some type of asynchronous communication between professionals and patients and those that referred to the improvement of the quality of care with repercussions on the efficiency of the health system. All study designs were included. They were restricted to Spanish, Catalan and English. The full review was conducted by an evaluator between April and May 2021. The search was conducted in a state of health emergency and oversaturation of primary care, so we focused on improving asynchronous communication between family doctors and patients.

### Study selection and data extraction

**Figure 1:**
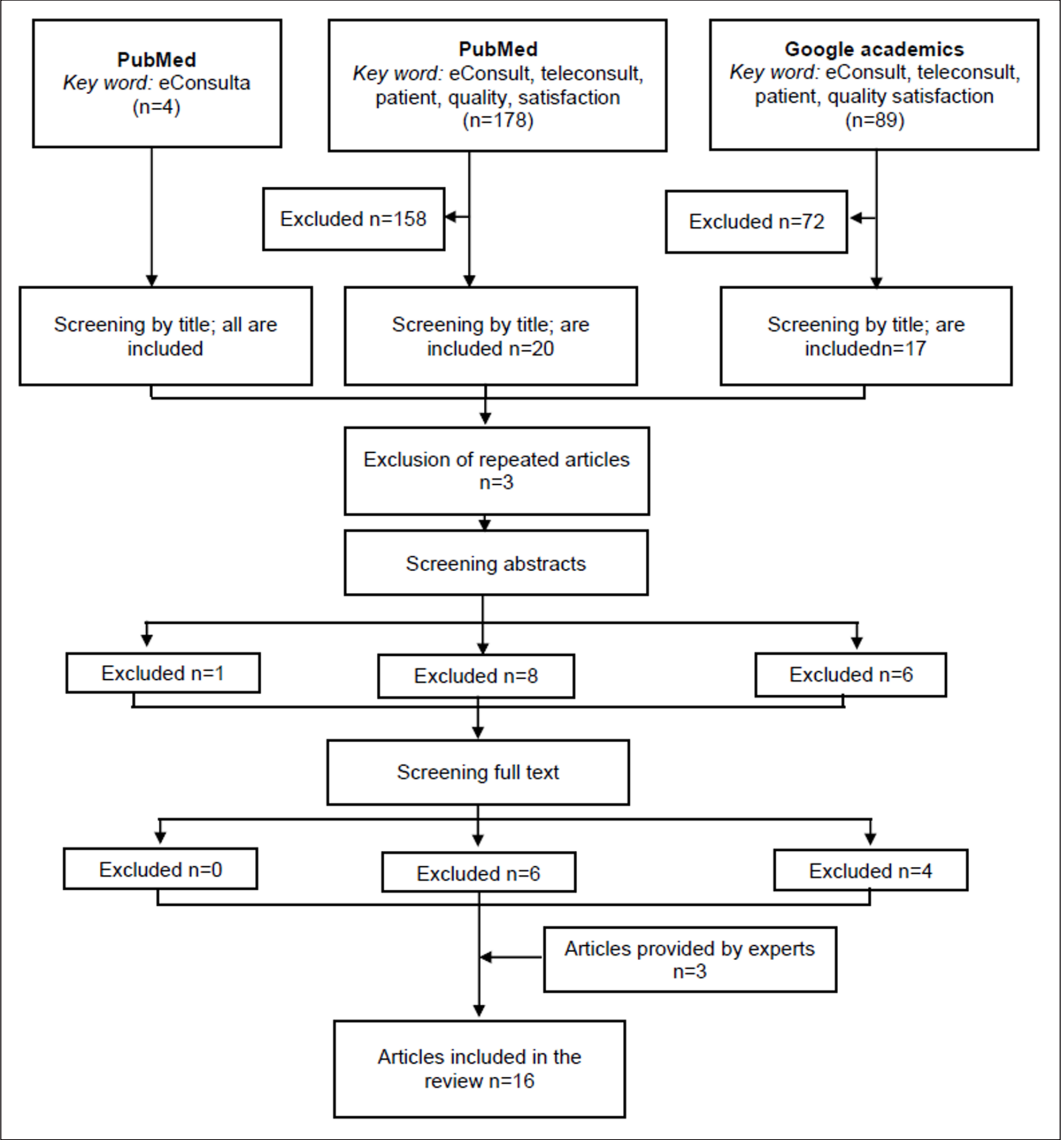
Flowchart of the selection process.

The first search in PubMed with the keyword “e-Consultation” alone yielded 4 results (Figure 1). In the second search in PubMed, all keywords were included and 177 results were obtained, of which 19 articles were finally included. In Google Academics, 89 results were obtained, including 19 articles. Repeated items were discarded. A review of abstracts was performed, discarding 1 from the first search, 8 from the second and 9 from the third. Then, in the full text review, six were discarded from the second search and four from the third search. The telemedicine experts of the Catalan Institute of Health in asynchronous teleconsultation provided 3 articles that were not yet published at that time. Annex A includes the main characteristics of included studies and excluded studies with reasons for exclusion.

## Results

### Characteristics of the included studies

A total of 16 studies, in Spain (n = 6), USA (n = 5), Canada (n = 3), Norway (n = 1) and one systematic review (n = 1) were included. Of the total, 7 are quantitative studies [1,3,13,14,15,16,17], 7 qualitative studies [11,18,19,20,21,22,23], and 2 mixed studies [24,25]. Finally, a qualitative analysis with deductive characteristics was carried out, identifying a total of 5 general themes: 1) Reduction of face-to-face visits 2) Services available and most frequent uses of asynchronous telematic consultation 3) Characteristics and perceptions of professional users 4) Characteristics and perceptions of users 5) Barriers and facilitators for the implementation of asynchronous telematic consultation.

### Reduction of face-to-face visits

Asynchronous telematic communications are one of the digital solutions with the capacity to reduce the number of face-to-face consultations in a primary care environment, promoting an improvement in the effectiveness and efficiency of a health system. One of the studies showed that 87.9% of the professionals considered that this tool was capable of resolving a consultation without the need for a doctor in person [13]. Seguí FL. et al. detected that the capacity of teleconsultation to avoid a face-to-face visit was relatively higher for consultations related to the management of prescriptions and the management of results and tests with a high consensus among professionals (67.95%) [1]. Also, a study in Spain showed that 70.88% of users avoided a trip to the primary care centre during the pandemic [17]. However, it should be noted that many face-to-face visits were transformed into teleconsultations in the pandemic context. The systematic review by Liddy C. et al. showed that 30 out of 43 studies reported a reduction in face-to-face visits, where the percentages were between 22% and 68% [20].

### Available services and most frequent uses of asynchronous telematic consultation

A study in Spain found that the most frequent uses were the management of test results (26.77%), management of repeat prescriptions (24.30%), and medical consultations (14.23%) with a high resolution rate [1]. Solans O. et al. compared the evolution of consultations carried out telematically from June 2018 to June 2020 in Spain, observing that the most significant increase since the beginning of the pandemic was in medical reports, followed by the results of blood tests [11].

A study in Norway showed different uses; firstly, appointment booking (66.4%), followed by prescription refills (54.3%) and finally, teleconsultation for administrative or logistical reasons [24].

### Features and perceptions of professional users

Solans O. et al. in Spain showed that the average age of professionals using asynchronous teleconsultation is 45 and 54 years old [3]. Also, Lee M. et al. described an age range of between 40 and 49 years [18]. The most recent study by Saigí-Rubió F. et al. confirmed that 33.4% of the professionals were between 40 and 49 years old, 29.2% between 50 and 59, only 15.8% were over 60 years old and 16.8% were under 30 years old [25].

Solans O. et al. observed that, in Spain, women used asynchronous teleconsultation in a higher percentage (67.70%) than men (30.70%) and in relation to the pandemic of COVID-19 [11], Saigí-Rubió F. et al. found that gender influenced intention to use in a post-pandemic scenario [25]. This was confirmed by the study of Liddy C. et al. in Canada, since out of a total of 2,052 teleconsultations 73.70% were women [15]. Lee M. et al. also showed that 68% were female [18].

Between 91% and 93% of practitioners surveyed in the Liddy C. et al. study reported high value to their patients and to themselves [15]. Likewise, the perceptions reported in Saigí-Rubió F. et al. coincided in the benefits of the use of asynchronous communications, for example, in the improvement in the relationship with patients and in the increase in the efficiency of their work [25]. Lee M. et al. showed how teleconsultations were generally more effective in coordinating efficient care, improving timeliness and quality of care [18]. Respondents to the Bishop TF. et al. survey in the United States commented that electronic communication provided convenient access to care, improved system efficiency, and saved patients time [19].

A study in Spain in 2021 showed how professionals’ perceptions of the degree of implementation of this tool were high [25]. Of the total, 44.9% indicated that telematic consultation had been fully implemented and 13.90% indicated that it had been implemented to a large extent. 38.60% mentioned that the pace of implementation was accelerated because of the current pandemic and 85.70% of the professionals agreed to continue using it after COVID-19.

On the other hand, other studies have reported that the perceptions of professionals with asynchronous communication were not so positive due to the increased workload and time with the consequent lengthening of the working day [18,19,21,23].

### User characteristics and perceptions

Polinski J. et al. in the United States observed in a survey of 1,734 patients that 70% of the users of asynchronous teleconsultations were women [14]. The review by Mold F. et al. and Zanaboni P. et al. also found that users were mainly women and used this service during working hours [23,24]. A recent study, comparing the characteristics of citizens who used asynchronous teleconsultation before and after COVID-19, reported that the majority of citizens who initiated a conversation through this digital solution were female in both cases (25.58% and 57.96% respectively) [11].

More disparate results were observed in the age of the patients using it. According to Polinski J. et al. they were younger profiles; 48% were in the 18–34 age range and, in contrast, Mold F. et al. showed that in some studies the users were younger and others reported a middle age [14,23]. A study in Spain comparing pre- and post-pandemic user profiles concluded that users were systematically younger after the onset of COVID-19 and that patients over 65 years of age were the least likely to use asynchronous telematic consultation [11]. In digital consultations in Norway there were more women among younger users and more men among older users [24].

Several studies reported a higher use of teleconsultations in patients of higher socio-economic status [3,14,23]. Zanaboni P. et al. observed that 59% of the users had university or higher education, which is related to a higher socio-economic profile [24].

One third (32%) of patients surveyed by Polinski J. et al. in the United States expressed a preference for receiving care via teleconsultation [14]. Solans O. et al. confirmed that users who were active at work did not usually go to the doctor, preferring a non-face-to-face visit [11].

In the survey of 1,734 patients in the United States, the majority stated they were very satisfied (94–99%), relating this to waiting time in the responses [14,23]. This conclusion has been confirmed by the study carried out in Spain, which detected a high percentage of satisfaction both in the pre-pandemic and during the pandemic [17]. Several studies showed that nearly 100% of patients reported that they would “definitely” or “probably” personally use it and recommend it to someone else [14,17,20].

In addition, users have reported a time saving of 10.5 minutes to refill the e-prescription compared to refilling by phone [19,24].

Mold F. et al. observed that e-consultation changed the doctor-patient relationship. The quality and safety of communication between groups potentially improved the therapeutic relationship, strengthening the relationship because of transparency, commitment, trust and shared decision making [23].

### Barriers and facilitators to the implementation of asynchronous telematic consultation

The most commented barrier in the literature was the increase in workload detected by the professionals, perceiving an increase in the responsibility associated with patient management and contributing to professional burnout [18,19,21].

Another barrier detected was the resistance to change on the part of the patient and the professionals. Bishop TF. et al. attributed patients’ resistance to change to inexperience with digital resources [19]. Different studies showed that the resistance to change of professionals was due to the lengthening of the working day, increased time load and changes in clinical responsibilities [18,19], although Bishop TF. et al. mentioned that practitioners reported a benefit in communication once they had used digital communication [19].

The most relevant facilitator found in the bibliography was the high degree of satisfaction in the use of the tool [13,14,23,24,15,16,17,18,19,20,21,22]. Seguí F. et al. observed that 64.96% of patients would have requested a face-to-face visit if they had not consulted via teleconsultation. In this way, a facilitator for implementation is the acceptance by users [1].

In Spain, one of the facilitators in the use of asynchronous telematic consultation was the outbreak of COVID-19. The use of this tool increased significantly and most professionals considered adopting this solution in their clinical routine [25]. Another study under review concluded that the pandemic possibly determined the definitive emergence of communication and information technologies in the Catalan health system [17]. Hilty D et al. found that teleconsultations generated a sense of immediacy and interaction, generating trust, while transcending physical materiality, time, space and direct consequences [22].

Another potential enabler is time savings [19,23,24]. In the study by Zanaboni et al. time savings was the most evident benefit for patients [24].

## Discussion

Different studies have reported that asynchronous telematic consultation produces a decrease in face-to-face visits. Waiting time for an in-person visit could be the trigger for using this tool. The results show that this digital tool has a variety of uses depending on the country where it is used. For example, in Spain it is mainly used for the management of tests performed, while in Norway there is no possibility of managing results through telematic consultation, and it is mainly used to request a face-to-face appointment. Thus, the results suggest that the study and analysis of the most frequent uses of remote consultation in each country can optimise the tasks that do not require presence, improving the quality of care, the management of tasks and patients and the effectiveness and efficiency in any health system [1,11,25].

It is a solution well appreciated by primary care professionals and patients, improving the effectiveness of consultations and access to care in an efficient way. The increased workload on professionals and the perceived difficulties in using this tool for patients and professionals may hinder its adoption and use [18,21,25]. All included articles have reported effectiveness and efficiency. In this way, each health system must evaluate the impact of this tool on both patients and professionals and generate strategies to facilitate its use.

Although the results have shown a high level of satisfaction in patients and professionals, consideration needs to be taken about the fact that the rapid spread of COVID-19 has maximized telematics consultation. Many professionals and patients who never had used this type of tool had to adapt quickly in order to stay in contact with the health care services. In this context, healthcare systems need to take into account that in the future, new digital health tools must be implemented with the involvement of all stakeholders, including patients [17]. The age of the patients who use the telematic consultation is variable and could suggest that the use of this tool is not linked to age but could be related to the digital resources of the users (digital literacy). The gender that uses this solution the most is the female gender [14]. Furthermore, the results have shown that telemedicine can improve the quality of care, enhance the patient experience and reduce healthcare system costs. Even so, this digital solution is not applicable for the whole population in general. Therefore, each health system has to assess the population profile in order to generate resources and tools based on the users of the system.

This study has several limitations, among them the limited bibliography found, since many studies were focused on specific pathologies. Even so, results from different countries have been integrated, improving the global vision of the implementation, the management of care flows and the use of asynchronous communications in a decentralised way.

Secondly, it should be taken into account that some articles included the COVID-19 pandemic period during its study time. This could be a bias as during the pandemic the reduction of face-to-face visits was forced and patients did not have a choice to do other types of visits.

Finally, is that the review and selection has been carried out by one person, and there may be a possible selection bias. In order to mitigate this potential bias, the full search was performed twice in different days to ensure that no relevant articles were excluded.

## Conclusions

Asynchronous remote communications between primary care professionals and patients in different countries and health systems have been analysed and it can be concluded that asynchronous remote consultations adapts to the demand of society. Even so, training of both professionals and patients is necessary. The inclusion of teleconsultations in routine medical practice should aim at improving workflow rather than increasing time and workload.

All in all, the high degree of satisfaction on both sides, the outbreak of COVID-19 and the effectiveness and efficiency of these asynchronous communications are key factors for their consolidation as tools for improving care management.
